# Amyotrophic Lateral Sclerosis (ALS) Awareness Month — May 2013

**Published:** 2013-04-26

May is Amyotrophic Lateral Sclerosis (ALS) Awareness Month. ALS, also known as Lou Gehrig’s disease, is a progressive, fatal, neurodegenerative disorder of both the upper and lower motor neurons. Persons with ALS usually die within 2–5 years of diagnosis. The etiology of ALS is not well understood, and currently there is no cure.

In October 2010, the federal Agency for Toxic Substances and Disease Registry (ATSDR) launched the National ALS Registry to collect and analyze data regarding persons with ALS in the United States. The main goals of the registry are to determine the incidence and prevalence of ALS within the United States, characterize the demographics of those living with ALS, and examine the potential risk factors for the disease. The registry uses data from existing national databases, including the Centers for Medicare and Medicaid Services and the U.S. Department of Veterans Affairs, as well as information provided by persons with ALS through a secure online web portal, available at http://www.cdc.gov/als. At the web portal, registrants can take brief online surveys regarding potential risk factors for the disease.

To achieve the registry’s goals, ATSDR is collaborating with the ALS Association (http://www.alsa.org), Muscular Dystrophy Association (http://www.als-mda.org), Les Turner Foundation (http://www.lesturnerals.org), and other organizations to make all persons with ALS and their families aware of the opportunity to register in the National ALS Registry. When sufficient data have been gathered to provide a representative picture of persons with ALS, ATSDR will begin analyzing the data and providing deidentified information so that researchers can gain a better understanding of the disease.

In addition to enrolling persons with ALS, ATSDR also has undertaken various initiatives to help strengthen the registry. These include implementing active surveillance activities to help evaluate the completeness of the registry in three states and eight metropolitan areas, funding a bioregistry feasibility study to link potential specimen data collected (e.g., blood, saliva, and tissue) with existing registry surveys, and funding external research on ALS risk factors and burden of disease. Additionally, ATSDR has launched a new research notification mechanism that puts researchers directly in contact with registry enrollees who are interested in taking part in new clinical trials and epidemiologic studies. Additional information regarding these initiatives and the National ALS Registry is available at http://www.cdc.gov/als.

